# Nurses' Trials—Responsibility

**Published:** 1887-03-12

**Authors:** 


### Words of Consolation.

[Communications for this column should be addressed, "Chaplain," care of the Editor of The Hospital, who will gratefully welcome any suggestions or inquiries from matrons, nurses, and the staff generally.]

### XXIV.—Nurses' Trials—Responsibility.

We are probably not far wrong in supposing that the burden of responsibility is one of the heaviest which some of our matrons and nurses have to bear. To undertake the tremendous responsibility involved in the care of the sick, and not to feel it, would indeed be a symptom of indifference or hardness much to be condemned. When we consider how very much, humanly speaking, the issues of life and death in hospitals depend upon the attention, activity, knowledge, and watchfulness of the attendants, we wonder how it is that more women do not break down under a sense of responsibility. There are some, of course, who do not feel responsibility as they ought, for want of a sufficiently exalted view of their calling. Some, thank God, regard their position in the best of all lights, feeling responsibility deeply, yet so resting (while they labour) in the Lord, as not to be oppressed. But others, again, are so oppressed by the feeling, that they find it a constant trial; and they are made unhappy every time they seem to have fallen below the standard of work or attention which is required of them. To these last, therefore, we venture to say, It takes two things to make a true calling to any office in life, namely, impulse and possibility. Many good women have felt strong impulses to devote themselves to nursing; but the possibility has not been open to them. Health, or family ties, or other circumstances had to be weighed in the balance against the impulse, and decided that the call was not really given. But, in your case, both the essentials to a true vocation seem to have been fulfilled. You not only felt the call, but have been able to shape your life accordingly, and to obey it. Has this no bearing upon the question of responsibility and its burdens? We think that it has very decidedly. He who gave the call foresaw and perfectly understood the responsibilities it would entail. Therefore He must be trusted implicitly to provide for the burden-bearer. We cannot always believe in apparently good impulses as coming from God, unless He opens a way for their performance. But when an impulse is given, such as you had, and then a door opened for its development in life, it may be the more certainly accepted as a definite calling. Now, if such is your view of a nurse's vocation, we may ask you as well to believe that He who called you more than shares your burden. Therefore, in a true exercise of faith you will not be unduly oppressed by it. It has been well remarked (by the author of *Amy Herbert*):—"Who are we, that we are to tremble at the prospect of any duty when God sees fit to appoint it? Have we indeed no help? Are we really left alone to wander, as best we may, through the intricacies of life's tangled wilderness, with no guiding post to direct us, no light to cheer us, no arm to uphold us? He who gave us the power of choice; He who made it so indestructible, that the most imperative human law, and the most abject spirit of weakness can never annihilate it, has He not promised His aid, His Spirit, Himself to be our Guide? The precious gift, which makes us living souls, and not machines, cannot be intended only as a snare to lead us to ruin."

This passage eloquently suggests a life of faith as the happy mean between the sin of not feeling responsibility, and the weakness of being overburdened by it.

(*To be continued.*)

### A GOOD DEFINITION.

A hospital is a house of Christian charity, where strangers are taken in as friends; where the sick are received as visitors; where experienced physicians and surgeons give the benefit of their skill; and where devoted women by kindly nursing, aid the recovery and soothe the sufferings of the patients.—H. B.

